# Altered Regional Homogeneity and Functional Connectivity during Microlesion Period after Deep Brain Stimulation in Parkinson's Disease

**DOI:** 10.1155/2021/2711365

**Published:** 2021-09-01

**Authors:** Bei Luo, Yue Lu, Chang Qiu, Wenwen Dong, Chen Xue, Dongming Liu, Li Zhang, Weiguo Liu, Wenbin Zhang

**Affiliations:** ^1^Department of Functional Neurosurgery, The Affiliated Brain Hospital of Nanjing Medical University, Nanjing 210029, China; ^2^Department of Radiology, The Affiliated Brain Hospital of Nanjing Medical University, Nanjing 210029, China; ^3^Department of Neurosurgery, The Affiliated Brain Hospital of Nanjing Medical University, Nanjing 210029, China; ^4^Department of Geriatrics, The Affiliated Brain Hospital of Nanjing Medical University, Nanjing 210029, China; ^5^Department of Neurology, The Affiliated Brain Hospital of Nanjing Medical University, Nanjing 210029, China

## Abstract

**Background:**

Patients with Parkinson's disease (PD) undergoing deep brain electrode implantation experience a temporary improvement in motor symptoms before the electrical stimulation begins. We usually call this the microlesion effect (MLE), but the mechanism behind it is not clear.

**Purpose:**

This study aimed to assess the alterations in brain functions at the regional and whole-brain levels, using regional homogeneity (ReHo) and functional connectivity (FC), during the postoperative microlesion period after deep brain stimulation (DBS) in PD patients.

**Method:**

Resting-state functional MRI data were collected from 27 PD patients before and after the first day of DBS and 12 healthy controls (HCs) in this study. The ReHo in combination with FC analysis was used to investigate the alterations of regional brain activity in all the subjects.

**Results:**

There were increased ReHo in the basal ganglia-thalamocortical circuit (left supplementary motor area and bilateral paracentral lobule), whereas decreased ReHo in the default mode network (DMN) (left angular gyrus, bilateral precuneus), prefrontal cortex (bilateral middle frontal gyrus), and the cerebello-thalamocortical (CTC) circuit (Cerebellum_crus2/1_L) after DBS. In addition, we also found abnormal FC in the lingual gyrus, cerebellum, and DMN.

**Conclusion:**

Microlesion of the thalamus caused by electrode implantation can alter the activity of the basal ganglia-thalamocortical circuit, prefrontal cortex, DMN, and CTC circuit and induce abnormal FC in the lingual gyrus, cerebellum, prefrontal cortex, and DMN among PD patients. The findings of this study contribute to the understanding of the mechanism of MLE.

## 1. Introduction

Deep brain stimulation (DBS) is a widely accepted surgical method used for the treatment of mid-to-late-stage Parkinson's disease (PD), which can significantly improve motor symptoms of PD and reduce drug-induced complications [1–3]. Currently, the subthalamic nucleus (STN) and internal pallidum (GPi) are commonly targeted by DBS in the treatment of PD [4–6]. After DBS electrodes are implanted, a temporary improvement in the motor symptoms of PD patients, including tremor, rigidity, and bradykinesia, can be observed immediately over several days to weeks. We usually call this the microlesion effect (MLE). Many studies have reported this [7–9]. However, the mechanisms underlying the improvement in motor symptoms are not well understood. In addition, previous studies have also suggested that postoperative verbal fluency [3, 10] and cognitive decline [3, 10] may be related to MLE. Some studies have found that MLE is likely associated with damage to peripheral neurons or glial cells, with subsequent degeneration loss, released neurotransmitters of local neurons, and edema around the electrode trajectory through the implantation of electrodes [7, 8].

Resting-state functional magnetic resonance imaging (rs-fMRI) is a type of brain MRI scan conducted while the subject remains motionless, closes his/her eyes, and does not carry out any active thinking in a calm state of awakening. Compared to the task state fMRI, rs-fMRI is simple and highly repeatable. It has been widely applied to investigate the alterations of brain activity in patients with neurological and mental illnesses [11–13]. The regional homogeneity [14] (ReHo) is a data-processing method proposed by Zang et al. in 2004 to analyze rs-fMRI data. The ReHo method indirectly reflects the spontaneous activity of neurons by analyzing the consistency of signaling fluctuations in the spatial adjacent voxel oxygen levels simultaneously using Kendall's coefficient of concordance (KCC) [14]. Reho has been widely used to study abnormalities in brain activity in PD [15–17] and can even be used as a potential imaging marker for PD [15, 18]. Conducting the ReHo method of the entire gray mass of the brain has almost shown test-retest reproducibility [19]. Functional connectivity (FC) is an fMRI data-processing method used to study brain networks to detect the correlations of spontaneous activity between different brain regions.

Currently, there are no literature reports on the use of ReHo, combined with the FC method, to study brain function during the postoperative microlesion phase of DBS. Therefore, the present study explored the functional changes of the brain region during the microlesion period by ReHo and FC analysis using the rs-fMRI whole-brain data.

## 2. Materials and Methods

### 2.1. Participants and Clinical Assessments

We initially recruited 34 PD patients for this study. All PD diagnoses were based on the UK Brain Bank Criteria for the diagnosis of PD in 1992 [20]. All PD patients met the indications for DBS surgery and underwent DBS surgery. The exclusion criteria included the following: (1) patients with a nonprimary Parkinson's disease diagnosis or an unknown Parkinson's disease diagnosis; (2) other central nervous system diseases or disorders (i.e., encephalitis, brain tumor, epilepsy); (3) drugs that affect brain function, such as antipsychotics and antidepressants; (4) contraindications for MRI scanning. In addition, 12 healthy controls (HCs) (six males and six females) with matched gender and age were recruited from the patients' family members and the community during the same period. All the subjects were right-handed. We used the Hamilton Anxiety (HAMA) and Hamilton Depression (HAMD) scales to evaluate the mental and psychological statuses of the participants. The overall cognitive level was assessed using the Montreal Cognitive Assessment (MoCA). The 39-item Parkinson's Disease Questionnaire (PDQ-39) was utilized to assess the quality of life in patients with PD. Furthermore, the patients' motor symptoms were assessed with the Unified Parkinson's Disease Rating Scale part-III (UPDRS-III). Verbal fluency (VF) was used to assess the patients' ability to produce words for a limited time and the patients' verbal memory. In this test, PD patients were asked to name as many animals as possible in 60 seconds, which is a semantic fluency test. Trail Making Test (TMT, part A, B) was used to evaluate the executive function of patients, and Benton judgment of line orientation test (BJLOT) was used to evaluate the visuospatial function of patients. In this study, the patients were tested using the above scales, including MoCA, UPDRS-III, VF, TMT, and BJLOT in three sessions: before DBS, one day after DBS, and one month after DBS. Preoperative PD data were included in the pre-PD group, and postoperative data were included in the post-PD group. The scale and MRI data of PD patients were collected 12 hours after drug disuse and before electrical stimulation to exclude the interference of drug and electrical stimulation to data acquisition. The study obtained the approval of the Ethics Committee of The Brain Hospital affiliated with Nanjing Medical University and written informed consent from all subjects.

### 2.2. Surgical Procedure

All PD patients who perform DBS surgery chose bilateral STN as the surgical target. Leksell planning system software was used to fuse the 3.0T head MRI scan data and CT scan data after the head positioning frame. The midpoint of the anterior commissure (AC) and posterior commissure (PC) is used as the origin to establish the coordinate system, and the surgical plan was made. The STN target was located 11–12 mm beside the origin, 3 mm backward, and 4 mm downward. In addition, the target coordinates were fine-tuned according to the specific position of the STN nucleus in the MRI image. The arc and ring angles of the implanted path were determined by designing a cranial approach that avoided cerebral surface vessels, sulci, and lateral ventricles. The operation was performed under a combination of local and general anesthesia. The preoperative plan was strictly followed during the electrode implantation, and the electrode position was not adjusted during and after the operation. DBS surgery was performed by a single neurosurgeon using a consistent surgical procedure. Postoperative CT and MRI reexaminations revealed no serious surgical complications except a small amount of intracranial gas in some patients.

### 2.3. Image Acquisition

MRI examinations were performed with a 1.5 Tesla GE Medical Systems scanner via an 8-channel head coil. The participants were asked to keep quiet, close their eyes, stay awake, and not think or carry out any voluntary movements on the examination bed during the scanning process. The patients were scanned before and after the first day of DBS, while the HCs group was scanned once. The imaging data were collected by a gradient-recalled echo-planar imaging (GRE-EPI) sequence with the following parameters: repetition time (TR) = 2000 ms, echo time (TE) = 40 ms, slice number = 28, thickness = 3.0 mm with no gap, flip angle (FA) = 90°, field of view (FOV) = 240 × 240 mm, matrix size = 64 × 64, voxel size = 3.75 × 3.75 × 3 mm^3^, and 128 total volumes. The T1-weighted data were collected by a 3D magnetization-prepared rapid gradient-echo (MPRAGE) sequence with TR = 11.864 ms, TE = 4.932 ms, FA = 20°, matrix size = 256 × 256, FOV = 152 × 152 mm, thickness = 1.4 mm, number of slices = 112, and voxel size = 0.59 × 0.59 × 1.4 mm^3^.

### 2.4. Data Preprocessing

The data preprocessing was performed using the rs-fMRI data-processing assistant (DPABI4.3, http://rfmri.org/dpabi) on the MATLAB 2013b platform (https://www.mathworks.com/products/matlab). The resting-state data were preprocessed using the following steps: (1) disregard the first five time points; (2) slice timing correction; (3) head motion correction (seven patients with a head translation >3.0 mm and rotation >3.0° in any direction were excluded); (4) spatial normalization to the standard Montreal Neurological Institute (MNI) template and resampled to 3 × 3 × 3 mm^3^; (5) removed the linear trend and time-bandpass filter (0.01 Hz *f* < 0.10 Hz). The nuisance variables, including 24 motion parameters, global signals, white matter signal, and cerebrospinal fluid signal, were regressed using the linear regression analysis. Spatial smoothing was conducted in preprocessing the FC analysis but not in ReHo analysis.

### 2.5. ReHo and FC Analysis

The filtering data without smoothing was utilized to conduct ReHo analysis in the DPABI software to calculate the consistency of each voxel and its adjacent 26 voxels in time series, which was the ReHo value of the voxel. The standardization of ReHo was obtained by dividing the KCC value of the individual voxel by the mean KCC of the whole brain. Finally, spatial smoothing was applied to the ReHo maps with a Gaussian kernel with full width at half maximum of 4 × 4 × 4 mm to further improve the image signal-to-noise ratio and decrease the errors caused by the spatial standardization process.

Brain regions with significant differences in ReHo values between the pre-PD group and post-PD group were defined as areas of interest (ROIs). FC were analyzed using a seed-based approach through the resting-state fMRI data analysis toolkit (http://restfmri.net/forum/REST). Finally, all FC maps are normalized by Fisher's z-transformation to improve the normality of data distribution.

### 2.6. Statistical Analysis

The two-sample *t*-test and chi-squared test were used to compare differences in demographic and clinical data between groups, respectively. In addition, the MoCA, UPDRS-III, VF, TMT, and BJLOT scores across different sessions in PD patients were compared using a repeated measures analysis of variance and post hoc tests were used between every two sessions. The statistically significant threshold was set to 0.05. All the above comparisons were performed using SPSS22 (Chicago, IL, USA) software.

The two-sample *t*-test was conducted to compare differences of ReHo value between the pre-PD group and HCs with age, sex, and mean framewise displacement (FD) as covariates in SPM12 (https://www.fil.ion.ucl.ac.uk/spm/software/spm12/). The data of the pre-PD and post-PD groups were rs-fMRI data obtained from the same group of patients at different time intervals. Therefore, the differences of ReHo/FC between the pre-PD and post-PD groups were assessed using paired *t*-tests with mean FD as a covariate. *P* < 0.001 at voxel level was used as the threshold of statistical significance. The threshold of statistical significance was set at *P* < 0.001. Multiple comparisons of the family-wise error rate with cluster *P* < 0.05 were applied to correct for excluding false-positive results.

## 3. Results

### 3.1. Demographic and Clinical Features

Twenty-seven patients (11 males and 16 females) were included in the PD group after excluding six patients, while 12 subjects (six males and six females) were recruited as the healthy controls HCs. The demographic and clinical characteristics of the subjects are presented in Table 1. PD patients and HCs did not differ in gender (*P*=0.59) and age (*P*=0.60). There was a significant difference in MoCA (*P* < 0.001), HAMA (*P* < 0.001), and HAMD (*P* < 0.001) between PD patients and HCs. The MoCA (*P*=0.001), UPDRS-III (*P* < 0.001), VF (*P* < 0.001), TMT (*P* < 0.001), and BJLOT (*P* < 0.001) scores for PD patients varied significantly over three sessions. In all the post hoc comparisons, we found significant differences (*P* < 0.001) between every two sessions of each scale except for MoCA before DBS and one month after DBS in PD patients (*P*=0.097).

### 3.2. Altered ReHo

**Figure 1:**
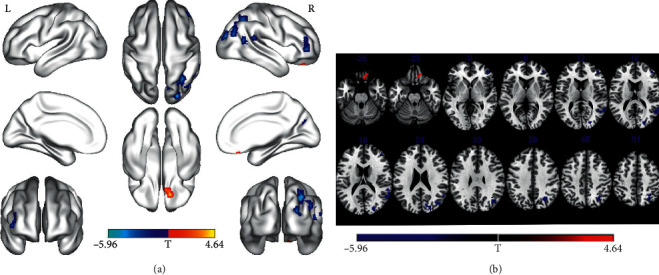
Regions showing ReHo differences between the pre-PD group and HCs (voxel *P* < 0.001, FWE correction with cluster *P* < 0.05). Regions showing increased ReHo in red and decreased ReHo in blue; surface (a) and transverse (b) views are shown; L, left hemispheres; R, right hemispheres; FWE, multiple comparisons of the family-wise error (voxel *P* < 0.001, cluster *P* < 0.05); ReHo, regional homogeneity; pre-PD, before DBS; post-PD, one day after DBS; PD, Parkinson's disease; DBS, deep brain stimulation; HCs, healthy controls.

**Figure 2:**
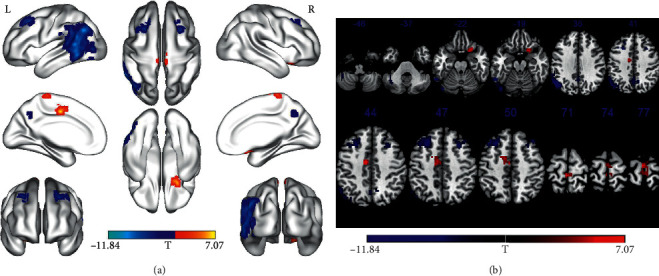
Regions showing ReHo differences between the pre-PD group and the post-PD group (voxel *P* < 0.001, FWE correction with cluster *P* < 0.05). Regions showing increased ReHo in red and decreased ReHo in blue; surface (a) and transverse (b) views are shown; L, left hemispheres; R, right hemispheres; FWE, multiple comparisons of the family-wise error (voxel *P* < 0.001, cluster *P* < 0.05); ReHo, regional homogeneity; pre-PD, before DBS; post-PD, one day after DBS; PD, Parkinson's disease; DBS, deep brain stimulation; HCs, healthy controls.

Relative to the HCs, the pre-PD group indicated increased ReHo in the right superior frontal gyrus (SFG), while they exhibited decreased ReHo in the right middle frontal gyrus (MFG), right middle temporal gyrus (MTG), right superior occipital gyrus (SOG), right middle occipital gyrus (MOG), and right angular gyrus (shown in Table 2 and Figure 1). After DBS, the post-PD group demonstrated increased ReHo in multiple brain regions, including the right inferior frontal gyrus (IFG), left supplementary motor area (SMA), left median cingulate and paracingulate (DCG) gyri, and bilateral paracentral lobule (PCL) compared to the pre-PD group. In addition, the post-PD group exhibited decreased ReHo in the left posterior cerebellar lobe (Cerebellum_crus2/1_L), left MTG, left angular gyrus, bilateral precuneus, and bilateral MFG compared to the pre-PD group (shown in Table 2 and Figure 2).

### 3.3. Altered FC

**Figure 3:**
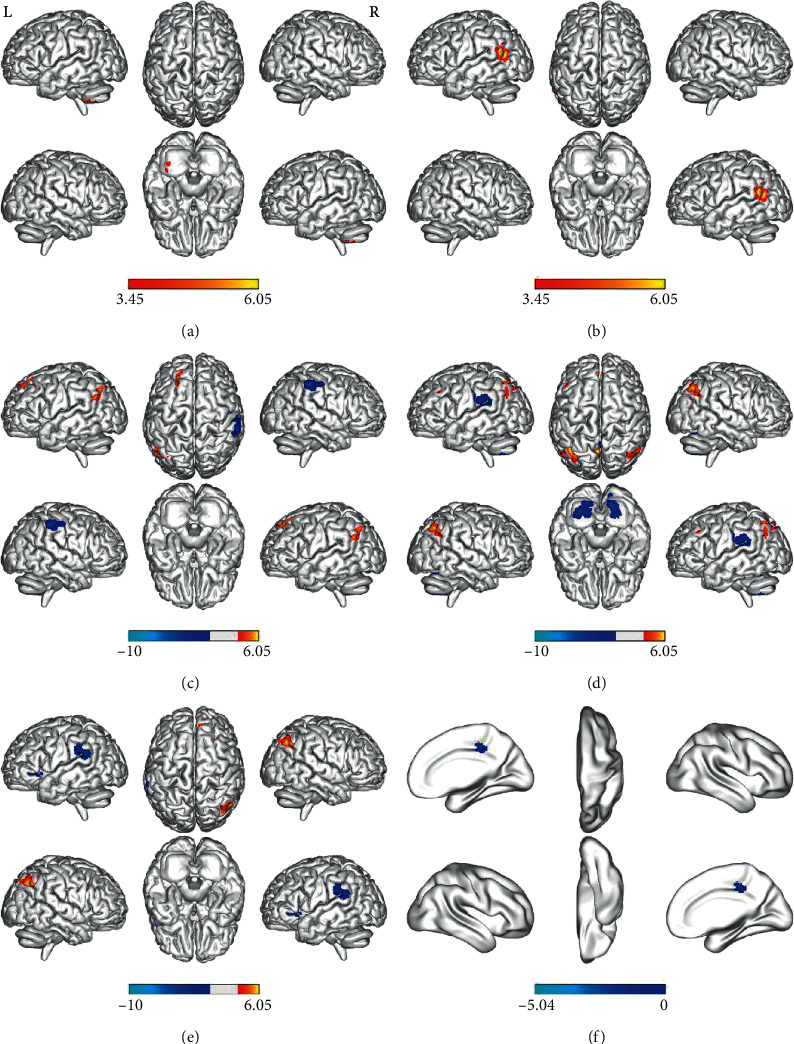
Regions showing altered FC between the pre-PD group and the post-PD group (voxel *P* < 0.001, FWE correction with cluster *P* < 0.05). Left posterior cerebellar lobe (a), left middle temporal gyrus (b), bilateral precuneus (c), left middle frontal gyrus (d), right middle frontal gyrus (e), and bilateral paracentral lobule (f) as areas of interest, and FC analysis was performed by the seed-based approach; regions showing increased FC in red and decreased FC in blue; L, left hemispheres; R, right hemispheres; FWE, multiple comparisons of the family-wise error (voxel *P* < 0.001, cluster *P* < 0.05); FC, functional connectivity; pre-PD, before DBS; post-PD, one day after DBS; PD, Parkinson's disease; DBS, deep brain stimulation.

Brain regions that showed significant differences in ReHo between the pre-PD and post-PD groups were eventually defined as ROIs. These brain regions include the right IFG, left SMA, PCL, left posterior cerebellar lobe, left MTG, precuneus, left MFG, and right MFG. Then we calculated the correlation between the seeds and the whole-brain voxel in a voxel-wise approach. There were no significant intergroup differences in FC for the two ROIs (right IFG and left SMA). The post-PD group demonstrated significantly reduced precuneus FC in the default mode network (DMN) (angular, precuneus, and superior frontal gyrus) compared to the pre-PD group. Similarly, we found reduced FC between the bilateral MFG and DMN. However, the post-PD group exhibited increased precuneus FC with right SMA and right supramarginal gyrus. In addition, the post-PD group had higher FC of left MFG with left supramarginal gyrus, bilateral cerebellum, and bilateral lingual gyrus. Furthermore, there was increased right MFG FC with left IFG and left supramarginal gyrus in the post-PD group. In addition, the post-PD group exhibited higher FC of PCL with right DCG compared with the pre-PD group. The above findings are presented in Table 3 and Figure 3.

## 4. Discussion

Herein, we used ReHo, combined with the FC method, to explore differences in brain function in resting state in PD patients during the microlesion period after DBS. The microlesion caused by implantation of the lone DBS electrodes can cause changes in the brain activity patterns and brain network. The increase or decrease in ReHo reflects increased or decreased consistency of spontaneous activity of neurons within the local brain region. Alterations in FC provide a basis for functional network reorganization of brain regions. After DBS surgery, the UPDRS-III score decreased significantly from 37.70 ± 12.04 to 27.52 ± 6.98, consistent with improved motor symptoms observed after surgery. After one month, the score deteriorated to 38.70 ± 10.89, reflecting the transient characteristics of symptom improvements during the microlesion period. In addition, on day one after surgery, the overall cognitive level, verbal fluency, visuospatial function, and executive function decreased according to the scale scores, suggesting that the microlesion of the implant pathway impaired these functions. One month after surgery, the VF performance, visuospatial function, and executive function were partially restored. However, the patients' overall cognitive level was also elevated to the preoperative level, suggesting that microlesion of the implant trajectory caused only a temporary decline in the patients' overall cognition, with no significant long-term impact, similar to a previous study. This study suggested that although STN-DBS selectively reduced frontal cognitive functions, it did not reduce patients' overall cognition or ability [21].

This study found that the ReHo value in the DMN-related region (right angular gyrus and right MFG) decreased in PD patients compared to HCs, suggesting that the DMN was partially impaired in PD, similar to results of previous studies [22, 23]. Previous studies reporting the brain region of alterations of ReHo in PD patients were not precisely consistent with this study, which might be attributed to the different subtypes and disease severity of PD patients included in this study. Many studies have consistently suggested that DMN plays an important role in cognitive processing [22–24]. Hou et al. [24] suggested that changes in DMN connectivity were characteristic of PD with a mild cognitive impairment. However, in a study on PD patients with unimpaired cognitive ability, the function of DMN was damaged, suggesting that the impairment of DMN had already occurred in PD patients before cognitive impairment [22]. In addition, a decreased ReHo value in the visual association cortex (right SOG and right MOG) was found in PD patients, consistent with prior imaging studies demonstrating reduced occipital leaf perfusion or metabolism in PD [25, 26].

We observed a higher ReHo value in the mesial premotor region (left SMA) and the primary motor cortex (M1), including in the bilateral PCL after deep brain electrodes implantation in the post-PD group relative to the pre-PD group, similar to the discovery of a previous study that focused on brain regions activated during the tapping test for microlesion [8]. SMA and M1 are important components of the basal ganglia-thalamocortical circuit. Some studies have consistently revealed that PD patients have less SMA activation or reduced activity [27–30], believed to be caused by damage to dopaminergic neurons in the basal ganglia of PD patients [31]. Haslinger et al. [28] reported that changes in SMA activity were highly associated with motor ability. After levodopa treatment, the activity of the SMA region increased, inducing relative normalization of impaired activation of the premotor cortex in PD patients [28, 31]. One study found that one-sided pallidotomy significantly increased the activity of SMA in PD patients during voluntary activity [32], consistent with the present study; however, the slight damage due to DBS surgery is less than that of pallidotomy.

Therefore, we believe that the increased activity of SMA after electrode implantation induces relative normalization of impaired mesial premotor regions in PD patients. The structure and function of SMA make this cortex region a possible target for neuromodulation therapy. M1 is the area responsible for motion output, and previous studies have demonstrated decreased M1 activity when PD patients exercise [33]. Kann et al. [34] reported a decrease in the volume of gray matter in the PCL and motor cortex associated with symptoms in the rigid motor subtype of PD. A previous study discovered decreased cortical thickness of PCL and motor cortex in PD patients with gait disorders [35]. Herein, it is believed that the increased ReHo of SMA and PCL in the microlesion period can relatively normalize the function of the motor-related brain areas and improve the neural activity of the basal ganglia-thalamocortical circuit, causing an improvement in postoperative symptoms in patients. The findings of this study are similar to those of a recently published paper [36]. Yue et al. used amplitude of low-frequency fluctuation (ALFF) to analyze the differences in brain activity before and after DBS surgery found that the ALFF value of the precentral gyrus decreased after surgery. ReHo revealed the neural synchronization of local brain regions activity, while ALFF reflected the spontaneous neural activity of local brain regions [16]. ALFF combined with the ReHo method can reflect the pattern of neural activity more comprehensively. The mechanism of surgical damage has been further elucidated by exploring the mechanism of MLE.

After DBS surgery, we observed decreased ReHo in DMN-related areas (left angular gyrus, bilateral precuneus) and the CTC circuit (Cerebellum_Crus2/1_L). A previous study of microlesion also showed decreased activity in DMN-related brain regions after DBS [36]. However, they did not assess executive function or speech function after DBS. The DMN is a system that is active during rest and is closely related to cognition. Several studies have discovered that after STN-DBS implantation, the patients' cognitive aspects, particularly verbal fluency [3, 10], decrease, which is caused by MLE [3, 21, 37]. One study showed a continuous decline in VF performance in the case of nonelectrical stimulation, indicating that it is mainly due to surgical damage instead of electrical stimulation [38]. Meanwhile, the post-PD group showed decreased ReHo values in bilateral MFG relative to the pre-PD group. The MFG is a key region of executive function, involved in cognitive processing, emotional regulation, and working memory [39–41], consistent with the decline in cognitive level, VF performance, and executive function observed in patients after surgery in this study.

Surprisingly, further FC analysis in these regions revealed that the post-PD group had a similar decline with DMN-related area and prefrontal cortex compared to the pre-PD group. The results of the present study once again validate this hypothesis. In summary, MLE can cause a reduction in the activity and connectivity of brain regions in the prefrontal cortex and DMN. Furthermore, there was increased FC of the precuneus and MFG with the following areas, including bilateral lingual gyrus, bilateral cerebellum, and right SMA. The lingual gyrus is a part of the occipital cortex and is involved in processing visual information [22]. However, after DBS, the visuospatial ability of patients decreased. We hypothesize that its enhanced connection to the DMN possibly represents a compensatory effect on visual processing in PD; however, further research is required. The cerebellum has an essential role in coordinating and controlling motion, and it is also a key area of the CTC. The decreased ReHo in the post-PD group might indicate partial damage to the CTC circuit after surgery. We speculate that in the microlesion stage, compared to the damage to the functioning of the CTC circuit, the damage caused by electrode implantation has a more significant compensatory effect on the function of the basal ganglia-thalamocortical circuit, ultimately causing an overall improvement in the patients' symptoms.

### 4.1. Limitations

First of all, this study was a preliminary study on the neural activity in the microlesion stage and the sample size is relatively small, so the sample size needs to be expanded for further study. Secondly, the period of postoperative microlesion is a phase of gradual change. This study did not conduct multiple assessments during the period of microlesion to better understand the changes in patients' symptoms. Finally, in this study, the effect of levodopa accumulation on the scale and resting-state data collection could not be completely eliminated even after withdrawal of anti-Parkinson/s disease drugs for 12 hours.

## 5. Conclusion

In conclusion, compared to HCs, alterations in DMN and visual association cortex areas of PD patients in this study further validated previous studies. The implantation of individual DBS electrodes changed the activity of the basal ganglia-thalamocortical circuit, prefrontal cortex, the DMN, and the CTC circuit and induced abnormal FC in the lingual gyrus, cerebellum, prefrontal cortex, and DMN in PD patients. However, the mechanism of microlesion remains to be further studied.

## Figures and Tables

**Table 1:** Demographic and clinical data of all subjects.

	HCs (*n* = 12), mean ± SD	PD (*n* = 27), mean ± SD	*P* value
Age (years)	63.92 ± 8.38	62.30 ± 8.97	0.60^a^
Sex (male/female)	6/6	16/11	0.59^b^
Disease duration (year)	NA	8.26 ± 2.78	—
LEDD (mg/d)	NA	765.78 ± 176.74	—
HAMA score	0.42 ± 0.51	5.59 ± 3.71	<0.001^a^^*∗*^
HAMD score	0.75 ± 0.87	5.63 ± 3.95	<0.001^a^^*∗*^
PDQ-39 score	NA	42.96 ± 16.97	—

*MoCA score*			
Before DBS	28.67 ± 1.07	24.22 ± 3.94	<0.001^a^^*∗*^
One day after DBS	NA	21.82 ± 4.78	—
One month after DBS	NA	23.78 ± 3.81	0.001^c^^*∗*^

*UPDRS-III score*			
Before DBS	NA	37.70 ± 12.04	—
One day after DBS	NA	27.52 ± 6.98	—
One month after DBS	NA	38.70 ± 10.89	<0.001^c^^*∗*^

*VF score*			
Before DBS	NA	20.63 ± 4.51	—
One day after DBS	NA	13.85 ± 4.10	—
One month after DBS	NA	16.30 ± 4.03	<0.001^c^^*∗*^

*BJLOT score*			
Before DBS	NA	21.30 ± 2.74	—
One day after DBS	NA	16.85 ± 3.21	—
One month after DBS	NA	19.48 ± 2.83	<0.001^c^^*∗*^

*TMT-A score*			
Before DBS	NA	45.70 ± 12.08	—
One day after DBS	NA	52.89 ± 11.62	—
One month after DBS	NA	49.96 ± 11.33	<0.001^c^^*∗*^

*TMT-B score*			
Before DBS	NA	99.07 ± 10.96	—
One day after DBS	NA	109.22 ± 10.93	—
One month after DBS	NA	103.74 ± 11.06	<0.001^c^^*∗*^

HCs, healthy controls; PD, Parkinson's disease; LEDD, levodopa equivalent daily dose; MoCA, Montreal cognitive assessment; HAMA, Hamilton anxiety scale; HAMD, Hamilton depression scale; UPDRS-III, the unified Parkinson's disease rating scale part-III; PDQ-39, the 39-item Parkinson's disease questionnaire; VF, verbal fluency; BJLOT, Benton judgment of line orientation test; TMT-A/B, trail making test part A/B; NA, not applicable; DBS, deep brain stimulation; SD, standard deviation. ^a^*P* value was obtained by a two-sample *t*-test between PD and HCs groups. ^b^*P* value was obtained by a chi-square test between PD and HCs groups. ^c^*P*value was obtained by a repeated measures ANOVA test in three sessions. ^*∗*^*P* < 0.05.

**Table 2:** Regions with ReHo differences between the pre-PD group and HCs, as well as between the pre-PD group and the post-PD group (voxel *P* < 0.001, FWE correction with cluster *P* < 0.05).

	Brain region (AAL)	Voxel size	MNI coordinate			Peak *T*-value
			*X*	*Y*	*Z*	
*Pre-PD* *>* *HC*						
Cluster1	Frontal_Sup_Orb_R	47	15	39	−30	4.6419
*HC* *>* *pre-PD*						
Cluster1	Frontal_Mid_R	39	45	42	15	−5.9629
Cluster2	Temporal_Mid_R	75	60	−54	12	−4.7052
Cluster3	Occipital_Sup_R	51	24	−87	18	−5.5474
Cluster4	Occipital_Mid_R	47	33	−60	27	−4.325
Cluster5	Angular_R	62	27	−66	42	−5.0054
*Pre-PD* *>* *post-PD*						
Cluster1	Cerebellum_Crus1/2_L	65	−39	−45	−45	4.8602
Cluster2	Temporal_Mid_L/angular_L	48	−60	−60	15	11.8391
Cluster3	Precuneus_R/L	88	0	−60	42	4.9435
Cluster4	Frontal_Mid_L	167	−27	24	51	7.2589
Cluster5	Frontal_Mid_R	62	39	30	54	5.6951
*Post-PD* *>* *pre-PD*						
Cluster1	Frontal_Inf_Orb_R	35	24	18	−21	−5.3848
Cluster2	Supp_Motor_Area_L/cingulum_Mid_L	51	−9	−9	45	−7.0682
Cluster3	Paracentral_Lobule_R/L	59	−3	−15	78	−5.5623

HCs, healthy controls; PD, Parkinson's disease; ReHo, regional homogeneity; FWE, the family-wise error; pre-PD, before DBS; post-PD, one day after DBS; AAL, anatomical automatic labeling; MNI, Montreal Neurological Institute; DBS, deep brain stimulation.

**Table 3:** Regions showing altered functional connectivities between the pre-PD group and the post-PD group (voxel *P* < 0.001, FWE correction with cluster *P* < 0.05).

Seed area	Brain region (AAL)	Cluster size	Peak MNI coordinate			Peak *T*-value
*Pre-PD* *>* *Post-PD*						
*Left posterior cerebellar lobe as the seed*						
Cluster 1	Cerebellum_8_L	196	−33	−48	−42	6.0512
	Cerebellum_Crus1_L					
*Left middle temporal gyrus as the seed*						
	Temporal_Mid_L	147	−60	−63	12	7.6778
*Bilateral precuneus as the seed*						
Cluster 1	Angular_L	198	−57	−60	30	7.0958
Cluster 2	Precuneus_L	93	−6	−57	36	5.4209
Cluster 3	Frontal_Sup_L	151	−21	36	48	6.239
*Left middle frontal gyrus as the seed*						
Cluster 1	Frontal_Sup_Medial_L	124	0	54	9	5.19
Cluster 2	Frontal_Mid_L	73	−45	30	33	4.8853
Cluster 3	Angular_L	302	−39	−63	48	7.1131
Cluster 4	Precuneus_L	214	−3	−66	45	6.8136
Cluster 5	Angular_R	236	45	−69	42	7.6871
Cluster 6	Frontal_Sup_Medial_R	201	−3	42	45	7.0057
*Right middle frontal gyrus as the seed*						
Cluster 1	Angular_R	221	51	−66	39	6.8844
Cluster 2	Frontal_Sup_Medial_R	140	18	45	42	5.054

*Post-PD* *>* *Pre-PD*						
*Bilateral precuneus as the seed*						
Cluster 1	Supp_Motor_Area_R	48	3	18	45	−5.0847
Cluster 2	SupraMarginal_R	53	60	−36	48	−5.1899
*Left middle frontal gyrus as the seed*						
Cluster 1	Cerebellum_8_L	141	−18	−78	−45	−5.5137
Cluster 2	Cerebellum_8_R	78	15	−78	−45	−4.9594
Cluster 3	Lingual_R	265	18	−63	−6	−7.4628
Cluster 3	Lingual_L	138	−18	−63	−6	−7.1345
Cluster 5	SupraMarginal_L	116	−54	−30	30	−5.4536
*Right middle frontal gyrus as the seed*						
Cluster 1	Frontal_Inf_Tri_L	52	−39	33	−3	−4.8022
	Frontal_Inf_Orb_L					
Cluster 2	SupraMarginal_L	45	−63	−33	30	−4.6723
*Bilateral paracentral lobule as the seed*						
Cluster 1	Cingulum_Mid_R	47	0	−33	42	−5.0405

Pre-PD, before DBS; post-PD, one day after DBS; AAL, anatomical automatic labeling; MNI, Montreal Neurological Institute; PD, Parkinson's disease; DBS, deep brain stimulation; FWE, the family-wise error.

## Data Availability

The relevant data used in this study are available from the corresponding author upon request.
